# Supplementary material for: Quantifying protocols for safe school activities
Source: PLoS One. 2022 Sep 14;17(9):e0273425. doi: 10.1371/journal.pone.0273425 (PMC9473403; doi:10.1371/journal.pone.0273425)
Supplement: Attachment — Submitted filename: Response to Reviewers_final.pdf [file pone.0273425.s002.pdf]

July 13th, 2022

Dear Editor,

We thank you and the referees for their constructive advice and the time dedicated to helping improve our manuscript. We addressed the concerns raised and improved the manuscript acoordingly.

Please find the reply point-by-point raised comments below.

Sincerely Yours

The Authors

Reviewer #1

This is an excellent research topic that investigates factors of COVID-19 virus transmission in schools using a variety of data sources. Findings from this study would be used as evidence-based to prevent COVID-19 in schools.

Thank you for your kind words and for recognizing the value of our work.

1. Paragraph 32-54 is not suitable/ appropriate in the introduction. The introduction should focus on study background rather than explaining methodology and summarising study findings.

We follow the referee suggestion while keeping lines 51-54 at the end of the introduction. As it serves as a hook for readers.

2. Results and discussion were combined in this article. Suggested separating according to IMRAD. I find it a bit mixed up here, and the authors need to discuss or elaborate more based on study findings.

We present classes of results, each based on different experiments and their peculiarities

I) Exploration of the efficacy of different protocols;

II) stability analysis of the protocols varying conditions for airflow and mask quality;

III) stability analysis of the protocols when faced with more infectious viral strains.

By combining the Results and the Discussion sections, we are able to present each class of results and immediately explores their consequences. This choice keep the focus of each class of results and highlight their individual consequences to the design of public health policies.

We hope to have clarified our reasons and that you can support this choice.

3. Multiple methods and analysis were used to yield study findings from this study. I would suggest there is a paragraph that lists out all the variables and explicitly explains the variables used before the methods in the analysis.

In Figure 2 we show the 4 types of parameters present in our model. Larger lists are still found throughout SI section 5 and the complete list is available in the repository.

Reviewer #2

I appreciate the importance of the results. I consider that the article has a high potential for impact.

We appreciate your evaluation of the importance of our work.

My main concern is in such analyses, it typically is possible to express the optimization problem in multiple ways with similar outcome. I think is very important to consider the deviation between the current practice and the solutions provided.

We agree that optimization depends sensitively on the constrains. In our study, optimization plays a role in the calibration of the epidemiological model from real world data. Then, our results do not optimize on the protocols to obtain a low number of infections. Rather, we analyse the outcome of multiple protocols that could be applied in schools.

> Introduction

- The introduction should avoid nominalizations.

- The introduction presents a summary of the project results, this can be deleted.

This has been corrected by removing lines 32-50. We also believe that striking out this denser part of the text reduces the nomizalization and improves the overall readability as you intended.

> Methods

- the methods have gaps in Agent based modeling

- The authors should be clearer about COMORBUSS.

We have improved on that in lines 112-135 and strengthened the link between the main text and the Supplementary Information.

> Discussion

- The authors should include the limitation of study.

Our largest limitation are the challenges involved in acquiring datasets of sufficient quality and their integration to COMORBUSS to produce good and reliable models. Following your suggestion, we present the most important problems in the main text (lines 243-354) and reinforced its integration to the SI.
